# Visual outcomes and subjective experience with three intraocular lenses based presbyopia correcting strategies in cataract patients

**DOI:** 10.1038/s41598-022-23694-9

**Published:** 2022-11-15

**Authors:** Meiyi Zhu, Wei Fan, Guangbin Zhang

**Affiliations:** 1 Department of Ophthalmology, Eye Institute and Affiliated Xiamen Eye Center of Xiamen University, School of Medicine, Xiamen University, No.336 Xiahe Road, Xiamen, 361001 China; 2 Fujian Provincial Key Laboratory of Corneal & Ocular Surface Diseases, Xiamen, 361002 Fujian China

**Keywords:** Eye diseases, Lens diseases

## Abstract

To compare the visual outcomes and subjective experience of three intraocular lenses (IOL) implant strategies. Retrospective comparative study. This study comprised patients who underwent phacoemulsification and bilateral implantation of extended depth of focus (EDOF) IOL (ZXR00; EDOF group), blended implantation of EDOF and bifocal IOL (ZXR00/ZLB00; blended group), and bilateral implantation of trifocal IOL (AT LISA tri 839MP; trifocal group). The outcomes included visual acuity (VA), visual defocus curve, contrast sensitivity, visual quality, quality of life, spectacle independence, and patient satisfaction. Follow-up was performed 3 months after the surgery. This study included 114 eyes of 57 patients (20 in EDOF group; 16 in blended group; 21 in trifocal group). Patients in the three groups had high quality of life, patient satisfaction, and good contrast sensitivity. The EDOF group had the worst near VA, but the visual quality was the best. The blended group had good VA and slight photic disturbance. The trifocal group obtained the best whole range of VA, but the photic disturbance was significantly severe than the EDOF group. Both the blended and trifocal groups achieved high spectacle independence, but some patients in the EDOF group need spectacle when dealing with close-range tasks.

## Introduction

Cataract patients hope to obtain a whole range of vision after the surgery, and the development of intraocular lens (IOL) innovation make this possible. Unlike monofocal IOL, which provide clear vision at a fixed distance only, presbyopia-correcting IOL enable patients to see objects at different distances.

The multifocal IOL is the main category of presbyopia-correcting IOL and has been widely used worldwide. It generates multiple foci by refractive or diffractive optical designs, produces a focused image and a defocused image on the retina simultaneously, and the brain suppresses the blurred images and selects the clearest image (called simultaneous vision)^1^. Although patients implanted with multifocal IOL can obtain higher spectacle independence than with monofocal IOL, the incidence rate of photic phenomena has been found to increase significantly^2^. Unpleasant photic disturbance is an important cause of patient dissatisfaction after the surgery^3^.

In recent years, a new category of presbyopia-correcting IOL has been applied in patients, namely the extended depth of focus (EDOF) IOL. The first available EDOF IOL in the marketplace was the Tecnis Symfony ZXR00, this design uses echelette technology to create a single elongated focus and combination with achromatic technology to improve visual quality^4^. A previous study indicated that this kind of IOL had similar image quality and halo effect to a monofocal IOL^5^ and provided excellent distance and intermediate visual acuity (VA)^6^. However, the near visual performance of EDOF IOL is unsatisfactory^4,7^. If patients want superior near vision, a micromonovision strategy^8^ or blended implantation with multifocal IOL (called contralateral implant strategy)^9–11^ would be a suitable alternative.

The contralateral implant strategy aims to combine the advantages of different multifocal IOLs to achieve good binocular visual performance and it is a less expensive way compared with trifocal IOL. The Tecnis Symfony ZXR00 design had a few limitations in near vision^4^ and it could be overcome by using a multifocal IOL in the contralateral eye to improve near visual performance^9–11^. Previous studies reported the satisfactory binocular VA from far to near distance after blended implantation of EDOF and diffractive bifocal IOL, with low incidence rate of photic disturbance and high patient satisfaction^9,11^.

The purpose of this study was to research the visual outcomes and subjective experience of patients who bilateral implantation of EDOF IOL, trifocal IOL, and blended implantation of an EDOF IOL with a bifocal IOL.

## Methods

### Patient population

This retrospective comparative study was approved by the Xiamen Eye Center affiliated with Xiamen University Ethics Committee, and the study adhered to the tenets of the Declaration of Helsinki. The informed consent had been obtained from all patients participating in the study. Fifty-seven patients (114 eyes) who underwent cataract surgery at Xiamen Eye Center, Affiliated Xiamen University, Xiamen, Fujian, China, between July 2021 and May 2022 were included in this study. The patients were divided into three groups: bilateral implantation of Tecnis Symfony ZXR00 IOL (EDOF group; 20 patients; 15 women and 5 men), blended implantation of Tecnis Symfony ZXR00 and Tecnis ZLB00 IOL (blended group; 16 patients; 6 women and 10 men), and bilateral implantation of AT LISA tri 839MP IOL (trifocal group; 21 patients; 14 women and 7 men). In the blended group, ZXR00 IOL was implanted in the dominant eye and ZLB00 IOL was implanted in the nondominant eye. The refraction target was emmetropia in the three groups. Patients with complications like capsular contraction syndrome, a dislocated intraocular lens, obvious posterior capsule opacification, and ocular fundus diseases, such as macular edema, during the follow-up were also excluded.

### Intraocular lens characteristics and surgical techniques

The AT LISA tri 839MP (Carl Zeiss Meditec AG, Inc.) is single-piece, aspheric (− 0.18 asphericity), diffractive trifocal lens. It has a 6.0 mm optic bench with a central trifocal zone over a diameter of 4.34 mm and a peripheral bifocal zone from 4.34 to 6.0 mm. The light distribution is 50%, 20%, and 30% for distance, intermediate, and near foci, respectively. The additions are + 3.33 D for near and + 1.66 D for intermediate at the IOL plane; in addition, it has a + 3.75 D add in its outer bifocal area. The corresponding additions projected on the spectacle plane are + 2.50 D (40 cm) and + 1.25 D (80 cm), respectively^12^.

The Tecnis Symfony ZXR00 (Johnson & Johnson Vision, Santa Ana, Inc.) is a single-piece, aspheric (− 0.27 asphericity) EDOF IOL. The optical zone is 6.0 mm. It has a patented diffractive echelette design to form an elongated focal zone with an addition of + 1.75 D at the IOL plane. The posterior achromatic diffractive surface has an echelette design for correction of chromatic aberrations and contrast sensitivity enhancement, which forms a step structure whose modification of height, spacing, and profile of the echelette extends the depth of focus^4^.

The Tecnis ZLB00 (Johnson & Johnson Vision, Santa Ana, Inc.) is a single-piece, aspheric (− 0.27 asphericity) bifocal lens. The optical zone is 6.0 mm. The IOL incorporates a posterior diffractive multifocal optic pattern designed to provide both near and distance vision, with a near power of + 3.25 D. This translates on the spectacle plane between + 2.50 D (40 cm) and + 2.75 D (36 cm)^13^.

A single experienced surgeon performed the phacoemulsification. The temporal clear corneal incision was about 2.2 mm. The size of the capsulorhexis was approximately 5.5 mm. All surgery was performed with a standard technique on an active-fluidics torsional phacoemulsification machine (Centurion Vision System, Alcon Laboratories, Inc.).

### Preoperative examination

The preoperative data collected included biomicroscopy, fundoscopy, uncorrected distance visual acuity (UDVA) at 5 m, corrected distance visual acuity (CDVA) at 5 m, pupil diameter and corneal spherical aberration (Pentacam; Oculus, Inc.), angle kappa (iTrace; Tracey Technologies Corp., Inc.), axial length and corneal astigmatism (IOLMaster 700; Carl Zeiss Meditec AG, Inc.). The IOL power was calculated using the Barrett Universal II formula.

### Postoperative examination

Follow-up was performed 3 months after the surgery. The postoperative examinations included: binocular and monocular UDVA at 5 m, uncorrected intermediate visual acuity (UIVA) at 80 cm, uncorrected near visual acuity (UNVA) at 40 cm, CDVA at 5 m, and manifest refraction; binocular and monocular defocus curves from + 1.0 D to − 4.0 D in decrements of 0.5 D were evaluated under distance correction; contrast sensitivity (CS); subjective outcomes included visual quality, quality of life, spectacle independence, and patient satisfaction.

The contrast sensitivity was assessed by the Binoptometer 4P^14^. The test was set at a virtual distance of 3 m and the luminance was set at 130 cd/m^2^. The optotype was Tumbling E (equivalent to a decimal visual acuity of 0.4). The contrast of the E letter was reduced gradually and the contrast levels were graded as 80%, 40%, 20%, 15%, 10%, 7.5%, 5%, and 2.5%. Patients were asked to identify the orientation of each E letter (5 Tumbling E for each set of contrast levels), if the patient recognized three optotypes correctly, the next level was tested. The recommend reference value was 15%, this level or lower can be regarded as normal CS^15^.

Visual quality was assessed by a questionnaire that was developed for this study. The information about the questionnaire was provided in the supplementary material. The optical phenomenon was evaluated in two ways: frequency and severity. The frequency was assessed by the following scale: never (100 scores), rarely (75 scores), sometimes (50 scores), most of the time (25 scores), and always (0 score). The severity was assessed by the following scale: none (100 scores), a little (75 scores), mild (50 scores), moderate (25 scores), and severe (0 score).

Quality of life was evaluated based on the Chinese version of the visual function index-14 (VF-12-CN)^16^. The difficulty scale was graded as not difficult (100 scores), a little (75 scores), moderate (50 scores), difficult (25 scores), and no reading anymore due to a vision problem (0 score). This questionnaire has 12 items, and the average score of each item is calculated separately (excluding the “not applicable” responses).

The patients were asked about their spectacle independence at far, intermediate, and near distances, graded as no need at all, occasionally need, and always need.

Patient satisfaction was assessed with a 5-point Likert scale: very satisfied (100 scores), satisfied (75 scores), neither satisfied nor dissatisfied (50 scores), dissatisfied (25 scores), and very dissatisfied (0 score).

### Sample size

The calculation of sample size was based on the main visual outcome: binocular uncorrected near visual acuity. In previous study, Lubin´ski et al. reported binocular uncorrected near visual acuity (40 cm) of patients bilateral implanted with AT LISA tri 839MP IOL was − 0.01 ± 0.04 logMAR and Tecnis Symfony ZXR00 IOL was 0.21 ± 0.15 logMAR^17^. To find a clinically significant difference between the three groups in our study, we used PASS 15.0.5 software to calculate the sample size based on the available data. The results showed that at least 10 patients were required in each group, the total number of 30 patients needed to be included in this study (alpha = 0.05 and power = 0.9).

### Statistical analysis

Statistical analysis was performed using SPSS for Windows software (v. 26.0, IBM Corp). The normal distribution of variable was evaluated using the Shapiro–Wilk test. Normally distributed variables were compared between the three groups using the One-way Analysis of Variance test. Non-normally distributed variables were compared between the three groups using the Kruskal–Wallis test. The chi-square test was used for the statistical analysis of the quantitative data. The One-way Analysis of Variance test was used to compare difference of age between the three groups. A *P*-value less than 0.05 was considered statistically significant.

## Results

The mean ages of the patients were 59.80 ± 7.26 years in the EDOF group, 61.69 ± 7.20 years in the blended group, and 59.33 ± 5.89 years in the trifocal group. No significant differences were found between the three groups (*P* = 0.556). The preoperative ocular characteristics are shown in Table 1.

**Table 1:** Descriptive measures for preoperative ocular characteristics of the EDOF group, blended group and trifocal group.

Measurement	EDOF group	Blended group	Trifocal group	*P* value
UDVA (logMAR)				0.083
Mean ± SD	0.63 ± 0.48	0.67 ± 0.41	0.49 ± 0.41	
Range	0.00 to 2.00	0.10 to 2.00	0.00 to 1.70	
CDVA (logMAR)				0.020
Mean ± SD	0.37 ± 0.44	0.41 ± 0.44	0.22 ± 0.33	
Range	0.00 to 2.00	0.10 to 2.00	0.00 to 1.70	
Corneal astigmatism (D)				0.053
Mean ± SD	0.77 ± 0.39	0.56 ± 0.30	0.65 ± 0.39	
Range	0.00 to 1.58	0.00 to 1.30	0.00 to 1.61	
Corneal spherical aberration (μm)				0.122
Mean ± SD	0.34 ± 0.11	0.32 ± 0.10	0.29 ± 0.12	
Range	0.17 to 0.66	–0.03 to 0.55	0.09 to 0.57	
Pupil diameter (mm)				0.093
Mean ± SD	2.73 ± 0.48	2.73 ± 0.66	2.90 ± 0.38	
Range	1.87 to 4.25	1.64 to 4.08	2.10 to 3.86	
Axial length (mm)				< 0.001
Mean ± SD	23.00 ± 0.94	24.08 ± 1.02	23.50 ± 1.15	
Range	21.46 to 25.55	22.33 to 26.24	21.30 to 26.04	
Angle kappa (mm)				0.058
Mean ± SD	0.31 ± 0.12	0.23 ± 0.11	0.26 ± 0.13	
Range	0.09 to 0.50	0.03 to 0.46	0.05 to 0.50	
IOL power (D)				0.003
Mean ± SD	22.45 ± 2.32	20.66 ± 2.35	21.25 ± 2.69	
Range	16.00 to 25.50	15.00 to 24.50	14.50 to 25.00	
Target refraction (D)				0.218
Mean ± SD	− 0.06 ± 0.18	− 0.08 ± 0.10	− 0.04 ± 0.10	
Range	− 0.59 to 0.17	− 0.24 to 0.16	− 0.17 to 0.17	

*UDVA* uncorrected distance visual acuity, *CDVA* corrected distance visual acuity, *logMAR* logarithm of the minimum angle of resolution, *D* diopters, *SD* standard deviation.

*P* < 0.05 means statistically significant difference between the three groups.

### Visual acuity and manifest refraction

**Figure 1:**
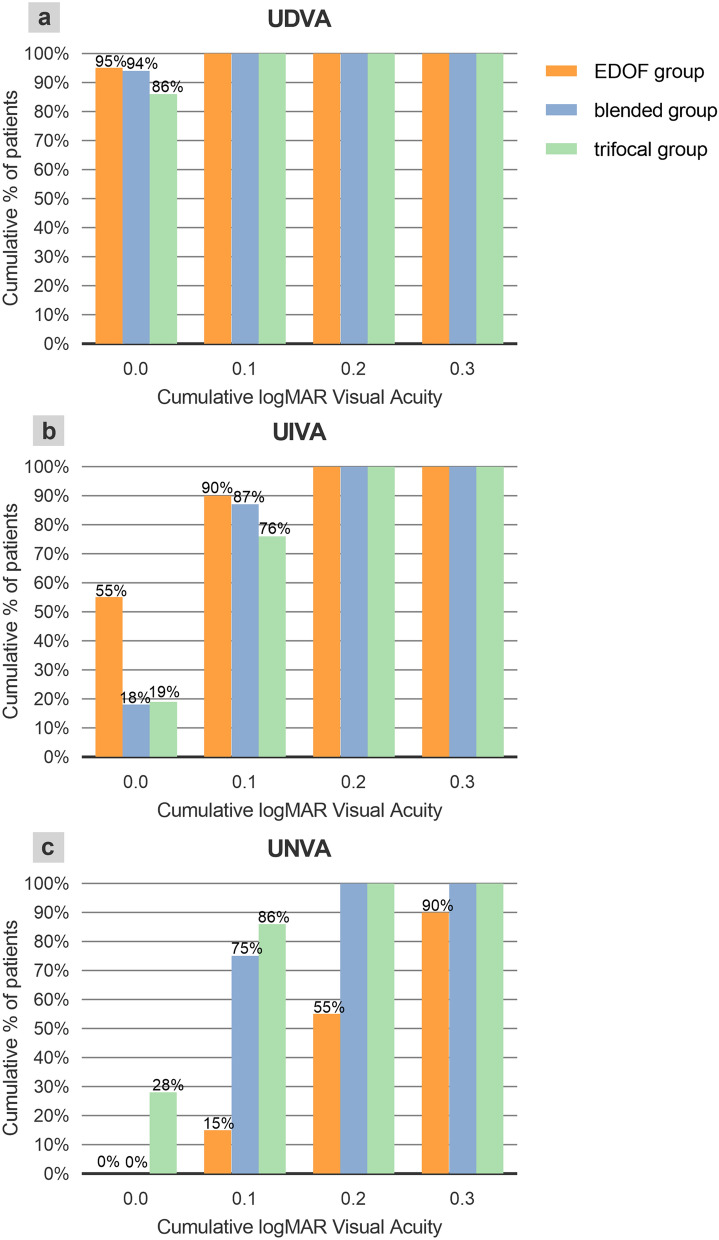
Cumulative distribution of UDVA, UIVA and UNVA for the EDOF group, blended group and trifocal group. (**a**) There were 95%, 94%, and 86% of patients in the EDOF, blended, and trifocal groups achieved UDVA 0.0 logMAR or better. (**b**) There were 55%, 18%, and 19% of patients in the EDOF, blended, and trifocal groups achieved UIVA 0.0 logMAR or better. (**c**) More than 70% of patients in the blended and trifocal groups achieved UNVA 0.1 logMAR or better, while 15% of patients in the EDOF group achieved 0.1 logMAR. *UDVA* uncorrected distance visual acuity, *UIVA* uncorrected intermediate visual acuity, *UNVA* uncorrected near visual acuity, *logMAR* logarithm of the minimum angle of resolution.

The mean binocular CDVA in the EDOF, blended, and trifocal groups were − 0.03 ± 0.05, − 0.02 ± 0.04, and − 0.03 ± 0.05 logMAR, respectively (*P* = 0.773). The mean binocular UDVA in the EDOF, blended, and trifocal groups were − 0.02 ± 0.04, − 0.00 ± 0.04, and − 0.01 ± 0.05 logMAR, respectively (*P* = 0.713). There were 95%, 94%, and 86% of the patients in the EDOF, blended, and trifocal groups achieved UDVA of 0.0 logMAR or better (Fig. 1 a). The mean binocular UIVA in the EDOF, blended, and trifocal groups were 0.05 ± 0.07, 0.09 ± 0.06, and 0.10 ± 0.07 logMAR, respectively (*P* = 0.041), and the trifocal group was statistically significantly worse than EDOF group (*P* = 0.048). There were 55%, 18%, and 19% of the patients in the EDOF, blended, and trifocal groups achieved UIVA of 0.0 logMAR or better (Fig. 1b). The mean binocular UNVA in the EDOF, blended, and trifocal groups were 0.23 ± 0.09, 0.12 ± 0.05, and 0.08 ± 0.07 logMAR, respectively (*P* < 0.001), and the EDOF group was statistically significantly worse than the blended group and the trifocal group (*P* = 0.002 vs. blended group; *P* < 0.001 vs. trifocal group). There were more than 70% of patients in the blended and trifocal groups achieved UNVA of 0.1 logMAR or better, while 15% of patients in the EDOF group achieved 0.1 logMAR (Fig. 1 c).

The mean monocular CDVA, UDVA, UIVA, and UNVA in the EDOF group was 0.00 ± 0.04, 0.02 ± 0.04, 0.11 ± 0.07, and 0.33 ± 0.15 logMAR, respectively. The mean monocular CDVA, UDVA, UIVA, and UNVA in the trifocal group was 0.01 ± 0.04, 0.04 ± 0.05, 0.14 ± 0.08, and 0.12 ± 0.08 logMAR, respectively. For the blended group, the mean monocular CDVA, UDVA, UIVA, and UNVA in eyes implanted with Tecnis Symfony ZXR00 was 0.01 ± 0.05, 0.03 ± 0.04, 0.10 ± 0.10, and 0.30 ± 0.14 logMAR, respectively; the mean monocular CDVA, UDVA, UIVA, and UNVA in eyes implanted with Tecnis ZLB00 was 0.02 ± 0.04, 0.02 ± 0.04, 0.22 ± 0.06, and 0.13 ± 0.06 logMAR, respectively; statistically significant difference was found between the two eyes in UIVA and UNVA (both *P* < 0.001).

The spherical equivalents in the EDOF, blended, and trifocal groups were − 0.08 ± 0.26, 0.00 ± 0.26, and − 0.05 ± 0.38 D respectively, with no statistically significant differences between the three groups (*P* = 0.543). Table 2 shows the postoperative binocular visual acuity and spherical equivalent of the three groups.

**Table 2:** Postoperative binocular visual acuity and spherical equivalent of the EDOF group, blended group and trifocal group.

Parameter	EDOF group	Blended group	Trifocal group	*P* value
UNVA (logMAR)				< 0.001
Mean ± SD	0.23 ± 0.09*	0.12 ± 0.05	0.08 ± 0.07	
Range	0.10 to 0.40	0.10 to 0.20	0.00 to 0.20	
UIVA (logMAR)				0.041
Mean ± SD	0.05 ± 0.07	0.09 ± 0.06	0.10 ± 0.07*	
Range	0.00 to 0.20	0.00 to 0.20	0.00 to 0.20	
UDVA (logMAR)				0.713
Mean ± SD	− 0.02 ± 0.04	− 0.00 ± 0.04	− 0.01 ± 0.05	
Range	− 0.08 to 0.10	− 0.08 to 0.10	− 0.08 to 0.10	
CDVA (logMAR)				0.773
Mean ± SD	− 0.03 ± 0.05	− 0.02 ± 0.04	− 0.03 ± 0.05	
Range	− 0.08 to 0.10	− 0.08 to 0.00	− 0.08 to 0.10	
SE (D)	− 0.08 ± 0.26	0.00 ± 0.26	− 0.05 ± 0.38	0.543

*UNVA* uncorrected near visual acuity, *UIVA* uncorrected intermediate visual acuity, *UDVA* uncorrected distance visual acuity, *CDVA* corrected distance visual acuity, *SE* spherical equivalent, *logMAR* logarithm of the minimum angle of resolution, *D* diopters, *SD* standard deviation.

*P* < 0.05 means statistically significant difference.

*means statistically significant difference compared with other groups.

### Defocus curves

**Figure 2:**
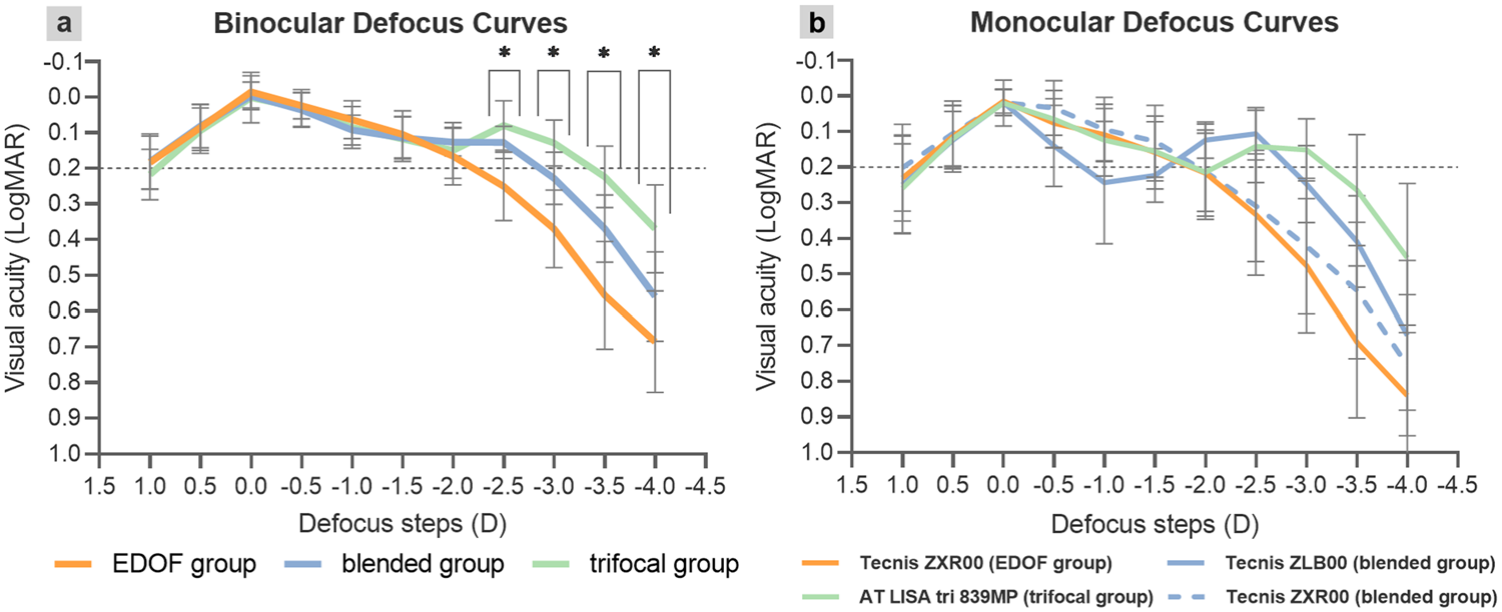
Binocular (**a**) and monocular (**b**) defocus curves of the EDOF, blended and trifocal groups. The binocular defocus range of the EDOF group, blended group and trifocal group was 2.5 D, 3.0 D, and 3.5 D, respectively. At vergence of − 2.5, − 3.0 and − 3.5 D, the binocular visual acuity of the EDOF group was statistically significantly worse than the blended group and trifocal group. At a vergence of − 4.0 D, the binocular visual acuity of the trifocal group was statistically significantly better than the EDOF group and blended group. All outcomes were compared between the three groups. Regarding the monocular defocus curves, the shape of curves showed the characteristic of AT LISA tri 839MP, Tecnis ZXR00 and Tecnis ZLB00 IOL design. Results are shown in logMAR notation, with reference to the 0.2 logMAR thresholds. ^*^means statistically significant difference between three groups. *logMAR* logarithm of the minimum angle of resolution, *D* diopters.

The binocular VA for each defocus step of the EDOF, blended, and trifocal groups are summarized in Table 3, and defocus curves are shown in Fig. 2(a). At a vergence of 0.0 D (distance vision), the three groups achieved similar VA results (*P* = 0.711). Over the vergence range of − 0.5 to − 2.0 D, all groups achieved VA better than 0.2 logMAR, with no statistically significant difference between the groups. At vergence of − 2.5, − 3.0 and − 3.5 D, the VA of the EDOF group was statistically significantly worse than that of the blended group and trifocal group (− 2.5 D: *P* = 0.002 vs. blended group,* P* < 0.001 vs. trifocal group; − 3.0 D: *P* = 0.013 vs. blended group,* P* < 0.001 vs. trifocal group; − 3.5 D: *P* = 0.030 vs. blended group,* P* < 0.001 vs. trifocal group). At a vergence of − 4.0 D, the VA of the trifocal group was statistically significantly better than that of the EDOF and blended groups (*P* < 0.001 vs. EDOF group; *P* = 0.004 vs. blended group).

**Table 3:** Binocular visual acuity at different defocus steps of the EDOF group, blended group and trifocal group.

Defocus steps (logMAR, mean ± SD)	EDOF group	Blended group	Trifocal group	*P* value
+ 1.0 D	0.18 ± 0.07	0.18 ± 0.08	0.22 ± 0.07	0.171
+ 0.5 D	0.09 ± 0.06	0.08 ± 0.06	0.09 ± 0.06	0.721
0 D	− 0.02 ± 0.04	− 0.00 ± 0.04	0.00 ± 0.07	0.711
− 0.5 D	0.02 ± 0.04	0.04 ± 0.05	0.03 ± 0.05	0.835
− 1.0 D	0.06 ± 0.05	0.09 ± 0.04	0.09 ± 0.06	0.166
− 1.5 D	0.10 ± 0.07	0.11 ± 0.06	0.12 ± 0.06	0.780
− 2.0 D	0.17 ± 0.08	0.13 ± 0.04	0.15 ± 0.08	0.278
− 2.5 D	0.25 ± 0.10	0.13 ± 0.05	0.08 ± 0.07	< 0.001
− 3.0 D	0.37 ± 0.11	0.23 ± 0.07	0.13 ± 0.06	< 0.001
− 3.5 D	0.56 ± 0.15	0.37 ± 0.09	0.22 ± 0.09	< 0.001
− 4.0 D	0.69 ± 0.14	0.56 ± 0.12	0.37 ± 0.12	< 0.001

*logMAR* logarithm of the minimum angle of resolution, *D* diopters, *SD* standard deviation.

*P* < 0.05 means statistically significant difference between the three groups.

Figure 2(b) depicts the monocular defocus curves of the EDOF, blended, and trifocal groups. In the EDOF group, the ZXR00 showed good vision from far to intermediate distance (VA better than 0.2 logMAR), but decreased sharply over the vergence of − 2.0 D. In the blended group, the visual performance of the eyes implanted with ZXR00 were similar with the EDOF group; regarding the eyes implanted with ZLB00, the defocus curve showed two peaks at far and near distance. In the trifocal group, the AT LISA tri 839MP achieved good VA over the vergence range of 0 D to − 3.0 D, with slightly worse at the vergence of − 2.0 D, and have a peak at near distance.

### Contrast sensitivity

Contrast sensitivity was divided into two grades: normal (CS ≤ 15%) and abnormal (CS > 15%). As shown in Table 4, 85.0% of the patients in the EDOF group had normal CS and CS no more than 25%, 81.3% of the patients in the blended group had normal CS and CS no more than 25%, and 81.0% of the patients in the trifocal group had normal CS and CS no more than 20%. The results showed that there was no statistically significant difference between the three groups (χ^2^ = 0.271, *P* > 0.99).

**Table 4:** Binocular contrast sensitivity of the EDOF group, blended group and trifocal group.

Binocular contrast sensitivity	Normal (CS ≤ 15%)	Abnormal (CS > 15%)	CS range (%)
EDOF group, n (%)	17 (85.0)	3 (15.0)	5–25%
Blended group, n (%)	13 (81.3)	3 (18.7)	7.5–25%
Trifocal group, n (%)	17 (81.0)	4 (19.0)	5–20%
χ^2^	0.271		
*P*	> 0.99		

*CS* contrast sensitivity.

*P* > 0.05 means no statistically significant difference between the three groups.

### Visual quality

**Figure 3:**
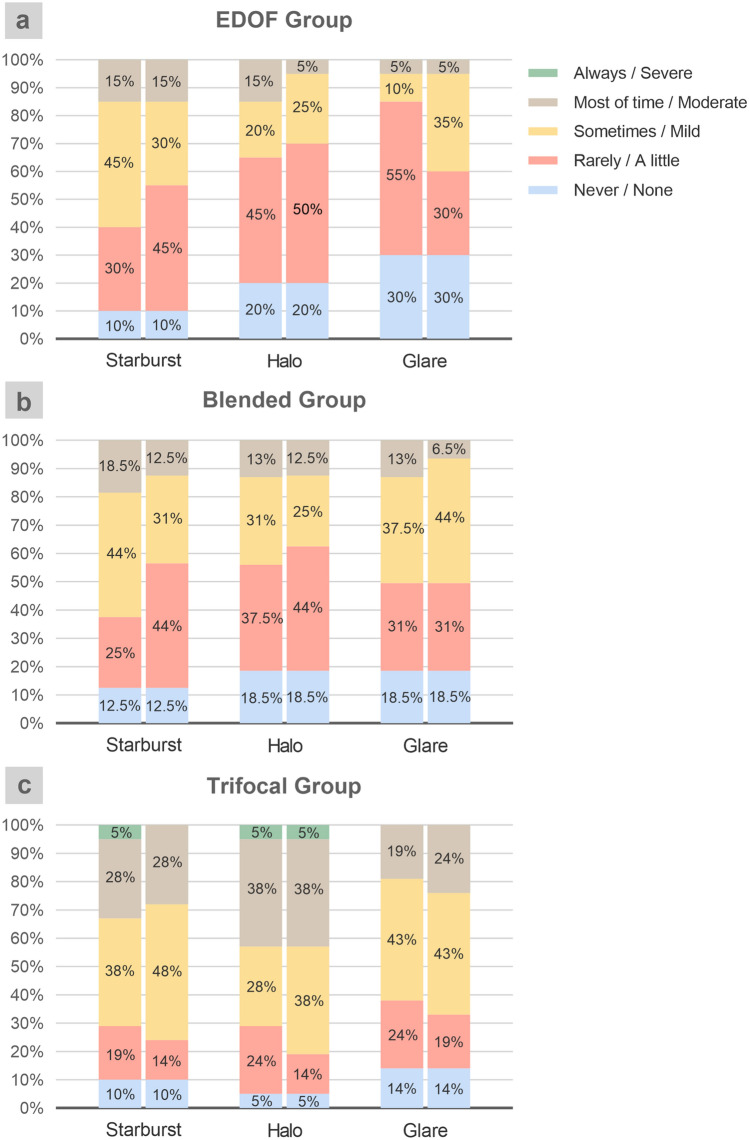
Results of the questionnaire regarding the perception of optical phenomenon in the EDOF group, blended group and trifocal group. The frequency and severity of starburst symptom in the EDOF group, blended group and trifocal group was not statistically significant different. The frequency and severity of halo symptom in the trifocal group was significantly severe than the EDOF group. The frequency of glare symptom in the trifocal group was significantly higher than the EDOF group. All outcomes were compared between the three groups. The first column of each optical phenomenon represents frequency, the second column represents severity.

Figure 3 shows the optical phenomena results of the questionnaire for the three groups. The mean scores of starburst frequency for the EDOF, blended and trifocal groups were 58.75 ± 21.88, 57.81 ± 23.66, and 50.00 ± 26.22, respectively (*P* = 0.448). The mean scores of starburst severity for the EDOF, blended and trifocal groups were 62.50 ± 22.21, 64.06 ± 22.30, and 51.19 ± 23.02, respectively (*P* = 0.120). The mean scores of halo frequency for the EDOF, blended and trifocal groups were 67.50 ± 24.67, 65.63 ± 23.94, and 46.43 ± 25.36, respectively (*P* = 0.021). A statistically significant difference was found between the EDOF and trifocal groups (*P* = 0.032), no statistically significant difference was found in the EDOF and blended groups (*P* > 0.99) and blended and trifocal groups (*P* = 0.095). The mean scores of halo severity for the EDOF, blended and trifocal groups were 71.25 ± 20.32, 67.19 ± 23.66, and 44.05 ± 23.59, respectively (*P* = 0.001). The severity of halo symptoms in the trifocal group was significantly severe than the EDOF group (*P* = 0.002) and the blended group (*P* = 0.018), with no statistically significant difference between the EDOF and blended groups (*P* > 0.99). The mean scores of glare frequency for the EDOF, blended and trifocal groups were 77.50 ± 19.70, 64.06 ± 24.10, and 58.33 ± 24.15, respectively (*P* = 0.025). A statistically significant difference was found between the EDOF and trifocal groups (*P* = 0.023), no statistically significant difference was found in the EDOF and blended groups (*P* = 0.231) and blended and trifocal groups (*P* > 0.99). The mean scores of glare severity for the EDOF, blended and trifocal groups were 71.25 ± 23.33, 65.63 ± 22.13, and 55.95 ± 24.88, respectively (*P* = 0.121).

### Quality of life

**Figure 4:**
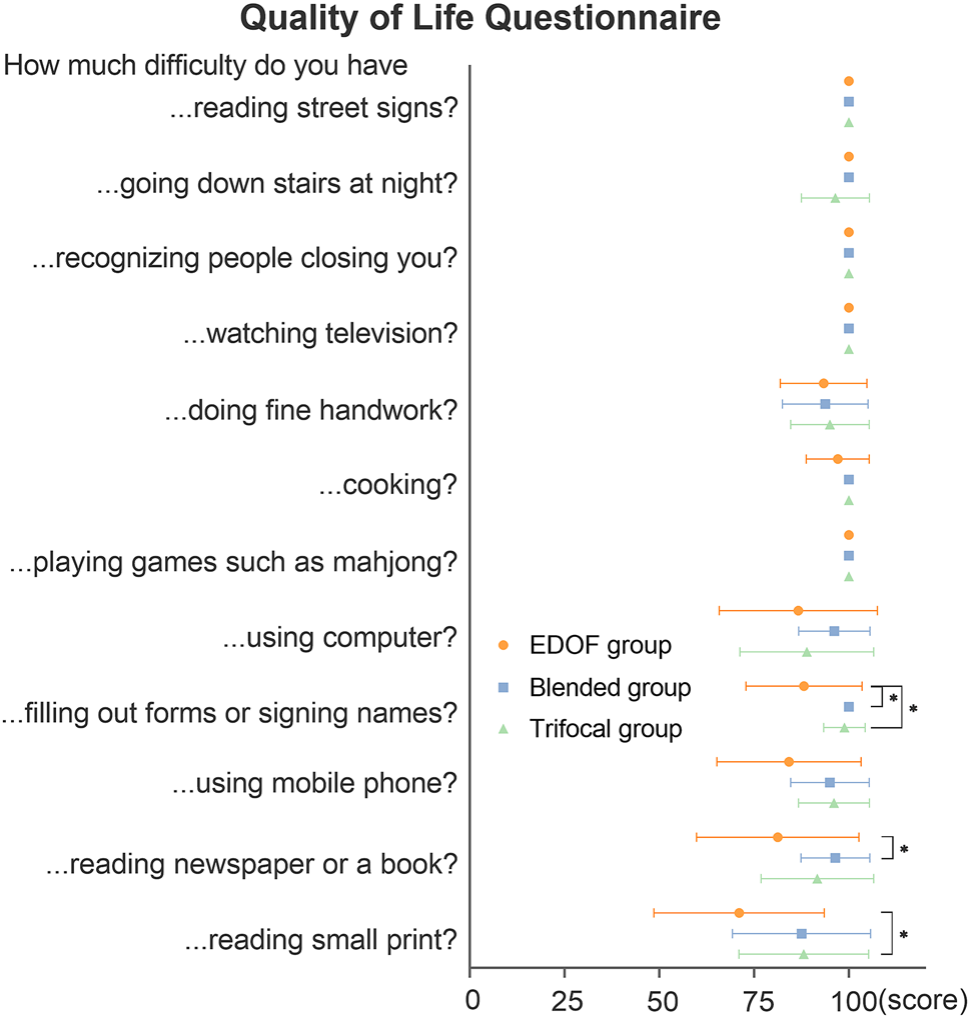
Results of the Chinese version visual function index-14 (VF-12-CN) for the EDOF, blended and trifocal groups. The patients of the EDOF group, blended group and trifocal group had no difficulty conducting far and intermediate distance activities. Patients in the EDOF group had more difficulty dealing with close-range tasks compared with the blended group and trifocal group. All outcomes were compared between the three groups. ^*^means statistically significant difference between groups.

Figure 4 shows the questionnaire items and the mean scores for each task. In far distance activities, the mean scores of the EDOF, blended, and trifocal groups were 100.00, 100.00, and 99.11 ± 2.24, respectively, with no statistically significant difference between the three groups (*P* = 0.069). In intermediate distance activities, the mean scores of the EDOF, blended, and trifocal groups were 94.48 ± 9.18, 97.14 ± 6.54, and 96.33 ± 7.06, respectively. No statistically significant difference was found between the three groups (*P* = 0.678). In near distance activities, the mean scores of the EDOF, blended, and trifocal groups were 81.67 ± 17.57, 94.66 ± 8.30, and 93.95 ± 10.18, respectively. The EDOF group was significantly worse than the blended group and trifocal group (*P* = 0.025 vs. blended group; *P* = 0.018 vs. trifocal group).

### Spectacle independence and patient satisfaction

The spectacle independence was more than 95% at the far and intermediate distances in the three groups. More than 90% of patients in the blended and trifocal groups achieved high spectacle independence at near distance, but that of only 60% of patients in the EDOF group (χ^2^ = 5.400, *P* = 0.026 vs. blended group; χ^2^ = 10.436, *P* = 0.005 vs. trifocal group). Regarding patient satisfaction, the mean scores of the EDOF, blended, and trifocal groups were 87.50 ± 15.17, 92.19 ± 11.97, and 91.67 ± 14.43, respectively, with no statistically significant difference between the three groups (*P* = 0.514).

## Discussion

Presbyopia-correcting IOL can provide significantly better near and/or intermediate VA than monocular IOL and maintain good distance VA in the meantime. Various types of presbyopia-correcting IOL have advantages and disadvantages. The trifocal IOL can provide good near, intermediate and far vision, but the IOL designed might lead to more photic disturbances^18,19^. The EDOF IOL can provide excellent distance and intermediate vision and high visual quality, but near VA is insufficient^4,7^. To the best of our knowledge, this is the first study to compare the visual outcomes and subjective experience of bilateral implantation of EDOF IOL (ZXR00), trifocal IOL (AT LISA tri 839MP) and blended implantation of an EDOF IOL (ZXR00) with a bifocal IOL (ZLB00) at the same time.

Our clinical findings showed that the three groups achieved good binocular distance and intermediate VA, which is consistent with previous studies^4,10,11,18^. The intermediate VA of the trifocal group (evaluated at the distance of 80 cm) was slightly worse than the EDOF group, which differs from the findings of Lubiński et al^17^. However, when comparing defocus curves, VA at vergences of − 1.0 to − 2.0 D (representing intermediate vision) was not statistically significantly different between the groups. When comparing near VA, the EDOF group was significantly worse than blended and trifocal groups. Similar to previous studies, the near visual performance of the ZXR00 was not good enough, this IOL design has limitations in near vision^6,7^. If the patient has a strong demand for near vision, the ZXR00 should not be used alone. The trifocal IOL showed good near vision, as reported in previous studies^12,18^. Meanwhile, the blended implantation of EDOF and bifocal IOL is an effective method for improving near VA^9–11^. Recently, micromonovision has been used to improve near VA when implanted with the ZXR00 IOL, but this approach may cause decreased far vision and additional unwanted photic disturbance from the low myopic eye^20^, therefore we did not use this method in patients.

The shape of defocus curves in this research was in agreement with previous studies^7,9,17^. It is worth noting that the VA at vergence range from − 2.5 to − 4.0 D of trifocal group was significantly better than the blended group. The reason may be the near vision mainly being provided by the nondominant eye in the blended group, the visual performance of nondominant eye was poorer than the dominant eye. Regarding the trifocal group, patients had near vision in both eyes, binocular VA is generally higher^21^. Moreover, the add power of AT LISA tri 839MP was slightly higher than ZLB00, which could be another reason.

Regarding the optical phenomena evaluated in this study, the halo and glare in the trifocal group were significantly higher than in the EDOF group. As previously reported, the number of diffractive steps was associated with halo symptoms^22^. The ZXR00 had a posterior achromatic diffractive surface and patented diffractive echelette design to form an elongated focal zone, this special IOL design can effectively reduce the occurrence of photic phenomena^4^. Comparing the blended group with the EDOF group, the overall halo and glare symptoms were similar. Although the patients in the blended group had one eye implanted with a bifocal diffractive IOL, the photic disturbance did not increase significantly. It seems that the nondominant eye has less impact on visual quality than the dominant eye^23^. In addition, the lower near addition power with the ZLB00 showed fewer photic phenomena^13^. For starburst symptom, the frequency and severity between the three groups were similar.

The patients in the three groups had no difficulty conducting far and intermediate distance activities. However, when conducting near activities, such as filling out forms, signing names, and reading newspaper and small print, the patients of EDOF group were significantly worse. The patients in the EDOF group also had lower spectacle independence when performing near activities. It is obviously that VA influences daily activities and quality of life^24^. Patients of the three groups achieved high satisfaction, although the patients in the EDOF group had significantly worse near vision.

This study has some limitations. One limitation is that the intermediate VA was only tested at 80 cm, but 60 cm or 66 cm is also commonly used working distance. The VA measured at various distances will be more helpful in assessing visual performance of patients. Although we had evaluated the defocus curves, the defocus VA values may not reflect the vision of real-life conditions completely. Another limitation is that we only assessed the photic phenomena by questionnaire to evaluate the visual quality of patients. The evaluation of objective visual quality such as halo perception and intraocular scattering is lacking. Nevertheless, the results of our study are still meaningful, the photic phenomena outcomes in this study can provide more references for the clinical application of these presbyopia-correcting IOLs.

In conclusion, all patients in this study had high quality of life, patient satisfaction, and good contrast sensitivity. Patients bilateral implanted with EDOF IOLs achieved the best visual quality, but near vision was deficient, blended implanted with EDOF and bifocal IOL had good VA and slight photic disturbance, and bilateral implanted with trifocal IOLs provided an excellent full range of vision, but was more likely to encounter photic disturbance.

## Supplementary Information

Supplementary Information.

## Data Availability

The data that support the findings of this study are available on request from the corresponding author. The data are not publicly available due to privacy or ethical restrictions.
