# Supplementary material for: Visual outcomes and subjective experience with three intraocular lenses based presbyopia correcting strategies in cataract patients
Source: Sci Rep. 2022 Nov 15;12:19625. doi: 10.1038/s41598-022-23694-9 (PMC9666430; doi:10.1038/s41598-022-23694-9)
Supplement: Supplementary file 1 — Supplementary Information. [file 41598_2022_23694_MOESM1_ESM.pdf]

Visual Outcomes and Subjective Experience with Three Intraocular Lenses Based Presbyopia Correcting Strategies in Cataract Patients

Meiyi Zhu[1,2], Wei Fan, MD[1,2], Guangbin Zhang[*], MD[1,2]

1. Department of Ophthalmology, Eye Institute and Affiliated Xiamen Eye Center of Xiamen University, School of Medicine, Xiamen University, Xiamen, China.
2. Fujian Provincial Key Laboratory of Corneal & Ocular Surface Diseases, Xiamen, Fujian 361002, China.

[*]: corresponding author

Corresponding author:

Guangbin Zhang[1,2]

1. Department of Ophthalmology, Eye Institute and Affiliated Xiamen Eye Center of Xiamen University, School of Medicine, Xiamen University, Xiamen, China. 2. Fujian Provincial Key Laboratory of Corneal & Ocular Surface Diseases, Xiamen, Fujian 361002, China.

No.336 Xiahe Road, Xiamen, China, 361001

E-mail: 386975604@qq.com

Telephone: 86-13599523368

First author:

Meiyi Zhu[1,2]

1. Department of Ophthalmology, Eye Institute and Affiliated Xiamen Eye Center of Xiamen University, School of Medicine, Xiamen University, Xiamen, China. 2. Fujian Provincial Key Laboratory of Corneal & Ocular Surface Diseases, Xiamen, Fujian 361002, China.

No.336 Xiahe Road, Xiamen, China, 361001

E-mail: 289656865@qq.com

Telephone: 86-13003991869

Co-author:

Wei Fan[1,2]

1. Department of Ophthalmology, Eye Institute and Affiliated Xiamen Eye Center of Xiamen University, School of Medicine, Xiamen University, Xiamen, China. 2. Fujian Provincial Key Laboratory of Corneal & Ocular Surface Diseases, Xiamen, Fujian 361002, China.

No.336 Xiahe Road, Xiamen, China, 361001

E-mail: 35080530@qq.com

Telephone: 86-15806011929

Questionnaire about Visual Quality

This questionnaire describes and asks you about 3 photic phenomena experience you might have with your vision. You will be asked if you experience any of them and asked to answer questions about:

- how often they occur;
- how severe they are at their worst.

There are pictures for the photic phenomena experience. These pictures may not be exactly what you experience. Choose the one that is most like what you see or experience.

QUESTION 1

1.1 How often did you experience starburst?

☐ Never ☐ Rarely ☐ Sometimes ☐ Most of time ☐ Always

1.2 Using the pictures below, rate how severe your worst experience was with starburst. These pictures may not look exactly like what you see, but are a guide to help you choose your answer.

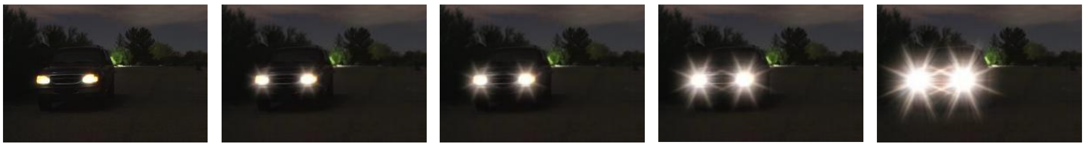

☐ None ☐ A little ☐ Mild ☐ Moderate ☐ Severe

QUESTION 2

2.1 How often did you experience halo?

☐ Never ☐ Rarely ☐ Sometimes ☐ Most of time ☐ Always

2.2 Using the pictures below, rate how severe your worst experience was with halo. These pictures may not look exactly like what you see, but are a guide to help you choose your answer.

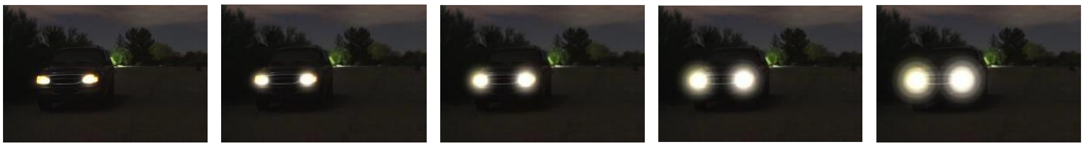

☐ None ☐ A little ☐ Mild ☐ Moderate ☐ Severe

QUESTION 3

3.1 How often did you experience glare?

☐ Never ☐ Rarely ☐ Sometimes ☐ Most of time ☐ Always

3.2 Using the pictures below, rate how severe your worst experience was with glare. These pictures may not look exactly like what you see, but are a guide to help you choose your answer.

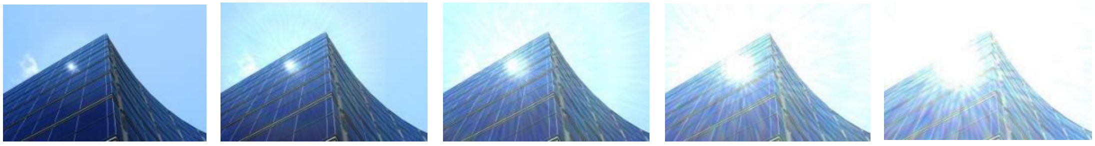

☐ None ☐ A little ☐ Mild ☐ Moderate ☐ Severe
